# Towards a “Sample-In, Answer-Out” Point-of-Care Platform for Nucleic Acid Extraction and Amplification: Using an HPV E6/E7 mRNA Model System

**DOI:** 10.1155/2012/905024

**Published:** 2011-12-22

**Authors:** Anja Gulliksen, Helen Keegan, Cara Martin, John O'Leary, Lars A. Solli, Inger Marie Falang, Petter Grønn, Aina Karlgård, Michal M. Mielnik, Ib-Rune Johansen, Terje R. Tofteberg, Tobias Baier, Rainer Gransee, Klaus Drese, Thomas Hansen-Hagge, Lutz Riegger, Peter Koltay, Roland Zengerle, Frank Karlsen, Dag Ausen, Liv Furuberg

**Affiliations:** ^1^NorChip AS, Industriveien 8, 3490 Klokkarstua, Norway; ^2^University of Oslo, 0316 Oslo, Norway; ^3^Department of Histopathology, Trinity College Dublin and Molecular Pathology Research Laboratory, Coombe Women and Infants University Hospital, Dolphins Barn, Dublin 8, Ireland; ^4^SINTEF ICT, MiNaLab Facility, Gaustadalléen 23C, 0373 Oslo, Norway; ^5^Institut für Mikrotechnik Mainz, Carl-Zeiss Straße 18-20, 55129 Mainz, Germany; ^6^Center of Smart Interfaces, TU Darmstadt, Petersenstraße 32, 64287 Darmstadt, Germany; ^7^Laboratory for MEMS Applications, Department of Microsystems Engineering-IMTEK, University of Freiburg, Georges-Koehler-Allee 106, 79110 Freiburg, Germany; ^8^BioFluidix GmbH, Georges-Koehler-Allee 106, 79110 Freiburg, Germany; ^9^Department of Micro and Nano Systems Technology, Faculty of Technology and Maritime Sciences, Vestfold University College, Raveien 197, 3184 Borre, Norway

## Abstract

The paper presents the development of a “proof-of-principle” hands-free and self-contained diagnostic platform for detection of human papillomavirus (HPV) E6/E7 mRNA in clinical specimens. The automated platform performs chip-based sample preconcentration, nucleic acid extraction, amplification, and real-time fluorescent detection with minimal user interfacing. It consists of two modular prototypes, one for sample preparation and one for amplification and detection; however, a common interface is available to facilitate later integration into one single module. Nucleic acid extracts (*n* = 28) from cervical cytology specimens extracted on the sample preparation chip were tested using the PreTect HPV-Proofer and achieved an overall detection rate for HPV across all dilutions of 50%–85.7%. A subset of 6 clinical samples extracted on the sample preparation chip module was chosen for complete validation on the NASBA chip module. For 4 of the samples, a 100% amplification for HPV 16 or 33 was obtained at the 1 : 10 dilution for microfluidic channels that filled correctly. The modules of a “sample-in, answer-out” diagnostic platform have been demonstrated from clinical sample input through sample preparation, amplification and final detection.

## 1. Introduction

Over the past decade, in a drive towards automation of laboratory testing, there has been considerable interest in developing more sensitive and specific molecular diagnostics with reduced time to result, in a way that is cost effective, requires minimal manual handling, and can be performed in a low-resource setting. In a recent review of “point-of-care” (POC) technologies, it was concluded that the most promising technology which fulfils this need is based on microfluidics, as it has the potential to control both the complex fluidic handling required during sample processing and the reagent mixing for nucleic acid amplification, in areas which are separated spatially and temporally, while being compatible with inexpensive materials and fabrication methods [1]. Microfluidic approaches in diagnostics achieve significant reagent volume reduction and thus cost-drive innovation, potentially achieving widespread penetration in non-hospital, non-specialised environments. However, microfluidic approaches are not without their challenges [2]. The field of “lab-on-a-chip” (LOC) diagnostics has grown rapidly from this basic need, and it is fast accelerating towards a “sample-in answer-out” platform for molecular diagnostics. A number of reviews have explored the potential use of LOC technology in areas such as clinical diagnostics, personalized medicine, global health, and forensics [1, 3–8].

While the level of complexity of LOC devices varies, the development of “sample-in and answer-out”, multifunctional, integrated LOC platforms is not beyond reach. Such a device which would facilitate the transfer of POC diagnostics to the near patient setting would have multiple positive outcomes for patient care. The potential introduction of POC-LOC technology to the doctor's office could dramatically reduce the time-to-result, facilitating early disease intervention and reduced patient anxiety. As generic nucleic acid technology can be adapted to a wide range of tests, a nucleic acid-based POC-LOC approach may facilitate a more complete and accurate diagnosis: however, it is not without its challenges, in particular, it must account for the enormous variation in source clinical material to obtain a valid result while also accounting for the heterogeneity that exists within a clinical sample type cohort.

The technology platform presented herein uses human papillomavirus (HPV) mRNA detection from cervical liquid-based cytology specimens as a model system. HPV is the oncogenic viral factor in cervical cancer [9]. Published data show that the incidence of cervical cancer is much higher in low-income countries than high-income countries [10]. As much as 83% of the cervical cancers occur in developing countries, which counts for 15% of female cancers. In developed countries, cervical cancer accounts for only 3.6% of new cancers. The mortality rates for cervical cancer are considerably lower than incidence as cervical cancer is highly preventable through cytological screening programs that facilitate the detection and treatment of precancerous lesions. Well-developed screening programs, especially in high-income areas, have contributed to a substantial decline for cervical cancer incidence and mortality. Introducing a platform as presented in this work for screening of precancerous lesions combined with effective treatment (test and treat possibilities) in low-income countries has enormous potential to reduce the mortality rate in these areas in the world [10, 11].

**Figure 1:**
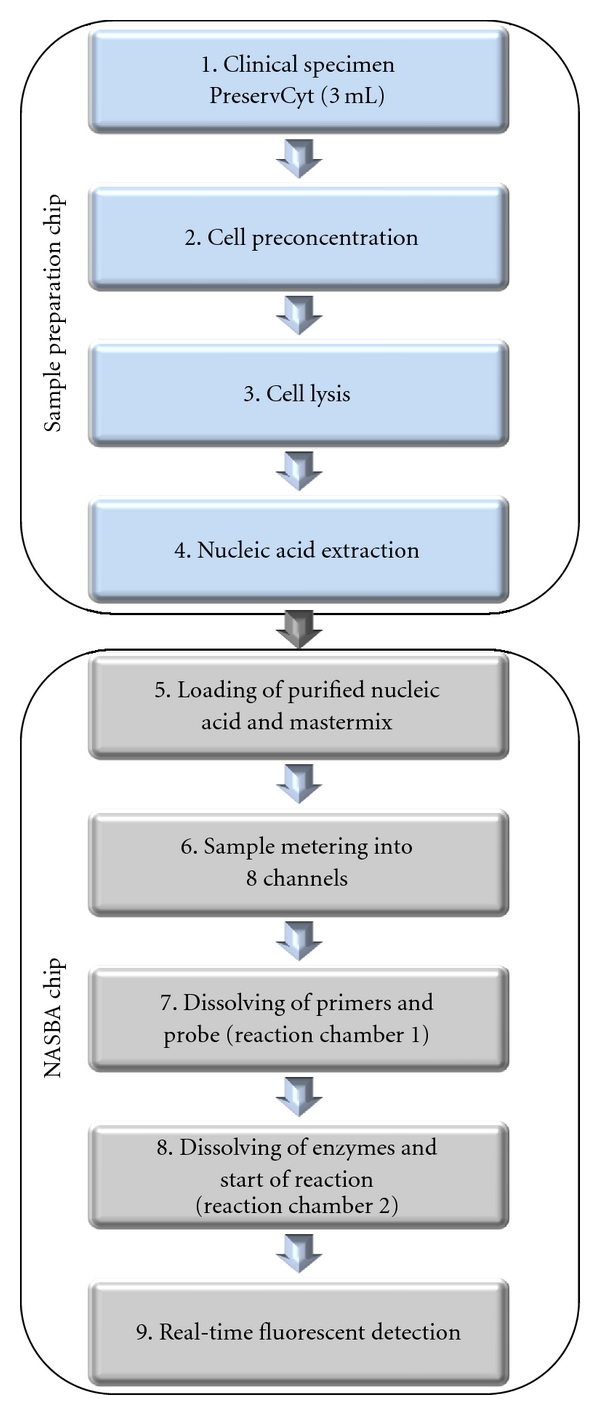
Schematic approach to the development of an integrated LOC device for the detection of HPV nucleic acid (involving a nucleic acid extraction chip and NASBA amplification chip).

The LOC modules described in this study comprises two distinct microfluidic chips with their associated supporting technologies, one for sample preparation and the other for nucleic acid amplification. At present, the modules may be used in tandem and will eventually be integrated together, achieving the “sample-in, answer-out” approach (Figure 1). Nucleic acid sequence-based amplification (NASBA) [12] technology with real-time fluorescence measurement was adapted to detect HPV mRNA in cervical specimens and cervical cell lines. The isothermal nature of NASBA greatly simplifies amplification strategies for nucleic acid detection on chip. This platform has huge potential within POC diagnostics as up to 16 different targets can be detected simultaneously for each clinical sample analysed. We present detailed information in relation to NASBA chip construction, microfluidics and results on analysis of cell line and clinical samples with the devices. We adopted an iterative approach to the chip development and optimisation of chemistries and assays and have used an industry gold standard (PreTect HPV-Proofer, NorChip AS, Norway) for HPV E6/E7 mRNA detection to compare our results. The standard PreTect HPV-Proofer was used to (a) determine the initial HPV status of the samples/cell lines and (b) to analyse the quality of the nucleic acid extract from the sample preparation chip. We outline some of the challenges involved in the creation of a fully integrated “sample-in and answer-out” LOC system.

## 2. Materials and Methods

### 2.1. Cell Lines and Clinical Specimens

For optimisation of both the sample preparation and the NASBA chip, total nucleic acid (TNA) extracted using the NucliSENS easyMAG instrument (bioMeriéux, France), from CaSki and SiHa cervical cancer cell lines obtained from the American Type Culture Collection (ATCC) were used. Nucleic acids were extracted from cell pellets containing 50,000–5 cell/mL [13]. For clinical evaluation, cervical smear specimens collected in PreservCyt, (Hologic Inc. Bedford, Mass, USA) and PreTect TM (NorChip AS, Norway) were used. Specimens with high-grade cervical intraepithelial neoplasia were chosen for “proof of principle” experiments. For gold standard HPV tests, total nucleic acid were extracted from 5 mL clinical PreservCyt specimen on Qiagen M48 BioRobot and eluted in 50 *μ*L. The baseline HPV mRNA status of specimens was determined for the 5 high-risk HPV types (HPV16, 18, 31, 33, and 45) and internal housekeeping control (U1A) using PreTect HPV-Proofer [11, 14–19]. Ethical approval for the study was obtained from the Research Ethics Committee at the Coombe Women and Infants University Hospital, Dublin, Ireland and the South African Ethics Committee at Pretoria Academic Hospital, Pretoria, South Africa.

### 2.2. Sample Preparation Chip

Cell lines and clinical specimens were extracted on a sample preparation platform which has been previously described elsewhere [13]. The sample preparation system is capable of performing total sample preparation and automated extraction of nucleic acids from human clinical specimens fixed in a methanol-based solution.

All necessary reagents for cell lysis, washing, and elution are stored on chip and the extraction is performed in 2 filter stages: one for cell preconcentration and the other for nucleic acid capture. The chip consists of COC (cyclic olefin copolymer; Ticona COC-5013) sealed with COP (cyclic olefin polymer; Zeon, Zeonor 1420R). All chips were fabricated by milling into blank injection moulded chips of size 64 mm × 43 mm × 3 mm. After mounting of the filters, the chips were solvent bonded with a 100 *μ*m COP foil and three turning valves were mounted on the chip surface. A customised design for the valve seals of the turning valves allows for the selective connection of channels on the chip.

**Figure 2:**
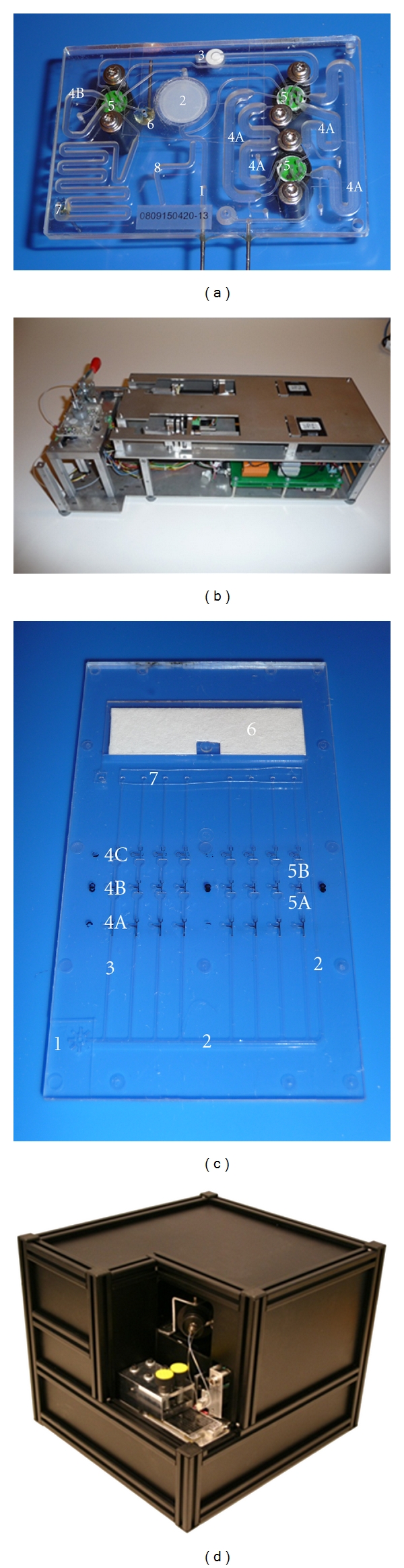
Automated LOC system for sample preconcentration, nucleic acid extraction, amplification, and real-time fluorescent detection. (a) Sample preparation chip: sample inlet [1], cell filter [2], a silica phase extraction filter [3], nucleic acid extraction reagent storage chamber [4A], storage chamber for DMSO and sorbitol [4B] turning valves [5], waste outlet [6], sample outlet [7], and a pressure sensor [8] (b) Sample preparation instrument (c) NASBA chip: sample inlet [1], supply channel [2], metering channels [3], valve 1 [4A], valve 2 [4B], valve 3 [4C], chamber 1 [5A], chamber 2 [5B], waste chamber [6], and instrument connection interface [7] (d) NASBA instrument.

The sample preparation chip (Figure 2(a)) consists of a sample inlet (1), cell filter (2), a silica phase extraction filter (SPE) (3), nucleic acid extraction reagent storage chamber (4A), storage chamber for DMSO and sorbitol (4B) turning valves (5), waste outlet (6), sample outlet (7), and a pressure sensor (8). For nucleic acid extraction, cells were collected and concentrated on a filter (Buckmann GmbH & Co. KG, Germany: Nylon, mesh width 10 *μ*m), where they were subsequently chemically lysed [13]. The released nucleic acid was captured downstream onto a silica filter (Genomed GmbH, Germany), in the presence of a chaotropic salt and extracted by solid-phase extraction, using a modified Boom's extraction method. Following extraction, downstream washing steps were performed to remove cellular debris. Subsequent air-drying was carried out before nucleic acid elution. Details of the reagents for lysis, wash, and elution have been previously published [13].

### 2.3. Sample Preparation Instrument

The sample preparation instrument (Figure 2(b)) has been described previously [13]. Briefly, it holds two modified syringe pumps containing two syringes: one to pump 3 mL sample through the cell capture filter and a second one for fluid actuation and drying by pressurised air. The latter is connected to an external 3-port/2way valve to allow for syringe reloading so that several piston strokes are possible, thus avoiding the need to draw air through the chip. Fluid control is achieved through 3 turning valves which are connected to motors below the chip holder table. A heater below the chip table elevates the temperature during lysis and for drying of the SPE filter before elution of nucleic acid. Custom built electronics addressed by a LabVIEW program automatically control all steps of operation from sample load to expulsion of the purified nucleic acid.

### 2.4. NASBA Chip

The NASBA chip consists of a disposable microfluidic cartridge composed of injection moulded COC (Topas 5013S-04 from Topas Advanced Polymers, Germany). The chips were manufactured by injection moulding using a Battenfeld, EM50/120 machine. The chip consists of a sample inlet (1), a supply channel (cross-section of 400 *μ*m × 200 *μ*m) (2), eight parallel reaction channels (3), 3 types of hydrophobic valves (4A, 4B, 4C), reaction chamber 1 (5A), reaction chamber 2 (5B), and a waste chamber (6) containing a highly absorbent filter paper (Figure 2(c)). Each of the parallel reaction channels consist of three parts; a metering channel (400 *μ*m × 120 *μ*m, volume of 740 nL) and two mixing/reaction chambers (volume approximately 800 nL). The two chambers and the metering channel are separated by hydrophobically coated capillary valves of increasing strength; the valve dimensions for the three valves 4A, 4B, and 4C are 200 *μ*m × 80 *μ*m, 125 *μ*m × 80 *μ*m, and 50 *μ*m × 50 *μ*m, respectively. Reaction chamber 2 serves as the real-time fluorescent detection chamber. The overall chip size is 75 mm × 44 mm × 1.5 mm. The chip surface was coated with a hydrophilic surface coating using 0.5% PEG (P2263, Sigma Aldrich, Norway) in methanol. The hydrophobic valves were coated with a mixture of 0.5% Teflon (Teflon AF 1600, Du-Pont, Germany) and 0.25% carbon black (type 901, Degussa, Germany) dissolved in perfluorinated solvent (Fluorinert FC-77, 3 M, Germany) and spotted using a PipeJet P9 dispenser (BioFluidix, Germany) [20]. After surface modification, a cotton linter filter (Schleicher & Schuell BioScience GmbH, Germany) was placed in the waste chamber, and the chips were manually sealed with polyolefin sealing foil containing microencapsulated glue on the contact side (HJ Bioanalytik GmbH, Germany).

### 2.5. The NASBA Reagents

The reagents used for NASBA chip reactions were obtained from the PreTect HPV- Proofer assay with the addition of 0.013 *μ*g/*μ*L BSA (Sigma Aldrich, Germany) for NASBA amplification. Premixed NASBA reaction mixtures were mixed according to the manufacturer's instructions. In order to proceed to the incubation temperature of 41°C, a prepreparation step was required. For the premixed reaction mixtures this involved incubating the master mix and purified nucleic acid sample together at 65°C for 2 minutes off chip. Subsequently, the temperature was reduced to 41°C and the enzyme was added to the reaction mixture prior to loading onto the NABSA chip.

For some analyses performed on the NASBA chip, reagent spotting on chip was conducted using the BioSpot liquid handling platform (BioFluidix GmbH, Germany) and the PipeJet dispenser [21]. The aluminium chip holder was cooled down to −80°C prior to the spotting to ensure freezing of the NASBA reagents upon impact within the chambers on chip. A volume of 125 nL of enzyme solution including 2% PEG was dispensed in reaction chamber 2 and 250 nL of the primer/probe mix was dispensed into reaction chamber 1. The freeze-drying procedure was performed in a commercial freeze-dryer (Triad, Labconco, USA). (The parameters for the freeze-drying procedure is described in the supplementary information available online at doi:10.1155/2012/905024.) In the prepreparation step for the NASBA chips containing freeze-dried enzyme and primer/probe mixes, only the remaining master mix reagents and the purified nucleic acid were mixed and incubated off chip at 65°C for 2 minutes. The temperature was reduced to 41°C prior to loading the reaction mixture on chip.

The reaction volume for PreTect HPV-Proofer was 20 *μ*L, while the reaction volume for NASBA on chip was 740 nL per reaction chamber.

### 2.6. The NASBA Instrument

Two versions of the NASBA instrument were developed for this study. The Uniplex Detector Version 1 of the instrument (Figure 2(d)) contains an optical unit, which has one excitation wavelength and one emission wavelength with the ability to detect HPV 16, 31, and 33. The instrument comprises two major units: a system for fluidic actuation and control and an optical detection unit.

The fluidic activation mechanism is operated by a pressure pulse generation system, consisting of a 500 mL pressure reservoir, three SMLD-5B valves from TechElan, a 0-1 PSID pressure sensor (Honeywell, Norway) for system feedback and a custom made syringe pump integrated into the instrument for pressure level adjustment. Sample heating and temperature control of 41°C and 65°C are achieved by a Peltier element (Marlow Industries, Sweden) and a thermistor (Elfa, Norway) for feedback is located beneath the chip surface. The accuracy of the heating system has been determined to be better than 0.1°C.

The optical detection unit consists of a fluorescence excitation detection module and a scanner. The scanner is a linear actuator (NEMA 17 linear actuator stepper motor from Ultra motion) which scans across the chip, halting above each reaction chamber to perform fluorescent measurements. The illumination fibre is a 600 *μ*m core diameter multimode fibre with numerical aperture of 0.22 (Thorlabs, Sweden). The detection fibre is a 1000 *μ*m core diameter multimode fibre with numerical aperture of 0.48 (Edmund Optics, UK). In the first generation instrument, (Uniplex Detector Version 1), a 1 W blue LED from Luxeon was used for excitation (excitation maximum wavelength 470 nm), and a band pass filter (Semrock, USA) was used to suppress unwanted wavelengths. The instrument has since been modified to include an additional amber LED (excitation maximum wavelength 597 nm) assessor (Luxeon) to facilitate multiple colour detection in the Multiplex Detector Version 2. During operation, the LED is modulated with a 310 Hz square pulse train with 50% duty cycle. The signal is demodulated by a digital lock-in amplifier, and integrated for 1 second. The amount of light emitted from the fibre end is close to 10 mW. At the detector side, two lenses (Edmund Optics, UK) were used to limit the angular cone through the emission filter (Semrock, USA). The modified instrument has additional emission filters. A Multi-Pixel Photon Counter (MPPC from Hamamatsu, Sweden) was chosen to serve as detector of the fluorescent signal. Excitation maximum wavelengths for the two fluorescent colours used, FAM and ROX, are 494 nm and 587 nm, with emission maximum wavelengths of 518 nm and 607 nm, respectively.

In addition to the above-described components, the instrument contains custom made electronics. The control of all instrument functions is performed *via* in-house software developed in LabVIEW (National Instruments).

## 3. Results

The overall objective of the system is to develop a hands-free diagnostic platform for detection of target nucleic acid in clinical specimens. The automated platform presented here performs chip-based sample preconcentration, nucleic acid extraction, amplification and real-time fluorescent detection with minimal user interfacing. It consists of two modular prototypes, one for sample preparation and one for amplification and detection. The prototype instruments with their associated disposable microfabricated chips (Figures 2(a) and 2(c)) have been tested individually; however, a common interface is available to facilitate later integration into one single setup.

The sample preparation system is capable of performing total sample preparation and automated extraction of nucleic acids from human clinical specimens fixed in a methanol-based solution. The automated sample preparation module [13] and early work on the amplification and detection module [20, 22–24], are presented elsewhere. In this paper, we present the combined sample preparation and detection platform in a “proof of principle” study with particular reference to testing from biological specimens and cell lines.

### 3.1. The Sample Preparation Chip and Instrument

This system has been described previously and tested on cell lines [13]. In this paper, we demonstrate its utility in clinical samples and its integration with the NASBA chip. Briefly, the cervical smear specimen was collected and concentrated on a filter, where it was subsequently chemically lysed. The released nucleic acid was then captured further downstream onto a silica filter in the presence of a chaotrophic salt solution and extracted by solid phase extraction using a variant of Boom's method [25]. Several washing steps were performed to remove the cell debris from the solid phase extraction matrix and after air drying the purified nucleic acid is eluted. The nucleic acid extracted is then transferred manually to the NASBA chip for amplification and detection.

### 3.2. The NASBA Chip and Instrument

**Figure 3:**
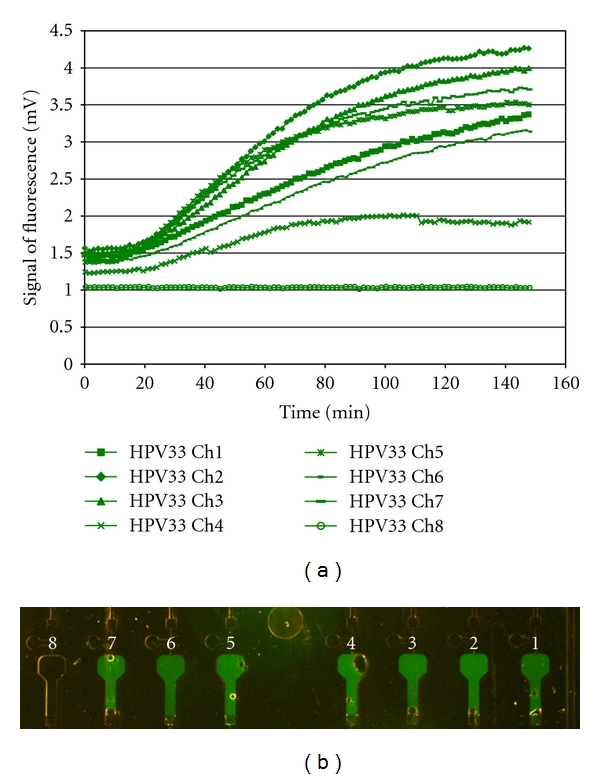
(a) The amplification plot of the clinical sample 508 (HPV 33, 1 : 10 dilution). All the 7 reaction chambers filled with sample are classified as positive using our inhouse data analysis code also used in the conventional PreTect Analyser instrument. Reaction chamber number 8 was not filled with sample; hence, no increase of fluorescence was observed. The offset of the curve is also lower than the filled chambers, as the autofluorescence of the reaction mixture was not present. (b) Fluorescent micrograph of reaction chambers (8–1) after amplification of clinical sample 508 (HPV 33, 1 : 10 dilution). The air bubble located in reaction chamber number 4 contributes to the lower fluorescent signal detected by the NASBA instrument. No sample has entered reaction chamber 8.

Nucleic acid extracted on the sample preparation chip is mixed with master mix and incubated at 65°C off chip, as described above. Initial experiments were performed on the NASBA chip using PreTect HPV-Proofer reagents premixed and added onto the chip with the nucleic acid. A further development of the NASBA chip involved freeze-drying primer/probes and enzyme onto the chip. Data on this is presented below. The mixture is loaded onto the chip inlet (Figure 2(c)), where it spontaneously is drawn into the supply channel by capillary forces. While the sample liquid fills the supply channel towards the waste chamber, the parallel metering channels are sequentially filled up to the position of the first capillary valve. When the sample reaches the waste chamber, it comes into contact with the filter paper which starts to absorb the liquid and hence acts as a capillary pump. The supply channel is effectively drained, leaving a precisely metered sample aliquot inside each of the metering channels. The pressure pulse system of the instrument is then actuated, transferring these sample plugs in parallel to the first reaction chamber. After a prescribed time, the pressure pulse system is again actuated transferring the sample plugs to the second reaction chamber, where the real-time fluorescence detection is initiated. The shape of the amplification curve generated by real-time increases in fluorescence determines whether the reaction is positive or not (Figure 3).

 Sample metering was evaluated in terms of the number of channels per chip successfully filled with sample during the biochemical and fluidic evaluations. The data is gathered from clinical tests using premixed reagents described below. In total, 16 chips were tested, corresponding to 128 metering channels. Of these channels, 102 were successfully filled with sample during the sample loading process, that is, a yield of 79.7%. Of the 16 chips tested, 6 were completely filled in all 8 channels.

Additionally, 9 NASBA chips were tested with freeze-dried enzymes deposited in reaction chamber 2 or the combination of freeze dried primer/probe mix deposited in reaction chamber 1 and enzymes in reaction chamber 2. In this case, 83.3% of the channels filled completely. Two chips filled all 8 channels. The unsuccessful filling of 20.3% and 16.7% of the metering channels may be due to clogging of the reaction channel, poor surface coating with PEG or Teflon overflow into the metering channel from valve 1 during spotting.

All on-chip flow control is performed *via* a single chip-to-world pressure interface. The chip and fluidic control system are designed such that the sample liquid is at no time in contact with the instrument. All on-chip fluidic control is achieved by pressurized air *via* the fluidic interface. In this way, the risk of cross-contamination between samples is avoided.

Flow actuation is achieved by generation of negative-amplitude pressure pulses delivered to the downstream end of the reaction channels *via* the connector block. During operation, the syringe pump and the feedback pressure sensor are used to set the desired pressure level in the reservoir. Once the correct pressure is reached, the sample plugs are transferred via hydrophobic valves on chip.

In order to detect the reactions in eight parallel chambers of the chip, the optical detection system consists of a fluorescence excitation/detection module and a scanner. The scanning function is realized mechanically by introducing an optical probe connected *via* flexible fibres to the illumination and detection source of the optical system. The probe is attached to a linear actuator and scanned across the chip, halting above each reaction chamber to perform the necessary fluorescence measurements. Two prototypes of the NASBA instrument were produced. The main difference of the two instruments is the optical features. The Uniplex Detector Version 1 detects one fluorescent colour, while the Multiplex Detector Version 2 detects two fluorescent colours.

### 3.3. “Proof of Principle”: The Sample Preparation Platform

The preliminary sensitivity and specificity data for cell line samples purified on the sample preparation platform was presented in Baier et al. 2009 [13]. The new data we present here refers specifically to the testing of clinical samples on the platform.

For evaluation of the sample preparation and nucleic acid extraction chip, cervical smear specimens collected in PreservCyt solution from 20 different patients were analysed. The HPV status of these specimens was as follows; HPV16 (*n* = 13), HPV33 (*n* = 3), coinfection HPV 16/33 and or 18 (*n* = 2), and negative (*n* = 2). From these 20 specimens a total of 28 extractions were performed to confirm the reproducibility of the system. Nucleic acid extracts from the sample preparation chip were then tested using the PreTect HPV-Proofer. We tested a range of nucleic acid extract dilutions, (1 : 1, 1 : 5 and 1 : 10). Overall, the detection rate for HPV obtained across all dilutions ranged from 50 to 85.7%. We did not observe a consistent pattern of amplification at any one dilution.

A subset of samples extracted on the sample preparation chip platform (*n* = 6) were chosen for complete validation on the NASBA chip platform using the criteria described in Section 2.

### 3.4. “Proof of Principle”: The NASBA Chip Platform

The preliminary sensitivity data generated on an earlier version of the NASBA chip platform showed detection limit of 20 cells/*μ*L for the SiHa cell line, which is comparable to the performance of PreTect HPV-Proofer [23]. The data we present here refers specifically to the testing of clinical samples on the NASBA chip platform which were processed for nucleic acids using the sample preparation platform.

In total, 16 NASBA chips were run using 6 clinical samples to evaluate reproducibility and to compare with PreTect HPV-Proofer as described above. All of the clinical specimens which amplified on PreTect HPV-Proofer did show positive amplification on NASBA chip (Table 1) in one or more dilutions of the TNA from the sample preparation platform.

Dilution of the nucleic acid extract 1 : 10 yielded a greater number of positive results on the NASBA chip than the undiluted extract and of the channels which filled correctly with the 1 : 10 dilution of extract, 69% were positive on the NASBA chip, compared with 37.7% of the channels correctly filled with the 1 : 5 dilution of extract. In 4 of the 6 samples tested, 100% amplification for HPV 16 or 33 was obtained at the 1 : 10 dilution for channels that filled correctly. An example of a positive amplification plot and fluorescent micrograph can be seen in Figure 3.

### 3.5. Further Developments towards a “Sample-In Answer-Out” Platform for Nucleic Acid Extraction and Amplification Using Freeze-Dried Reagents and Duplex Fluorescent Detection

**Figure 4:**
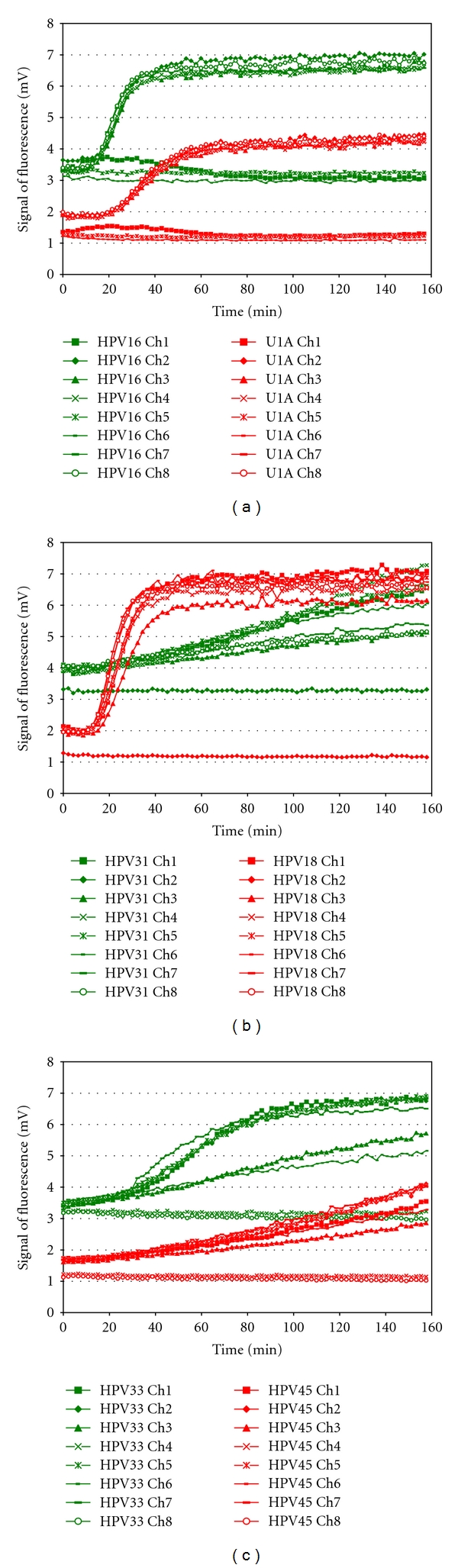
Two colour detection of fluorescence from NASBA amplified products using the Multiplex Detector Version 2 (a) Detection of HPV 16-FAM and U1A-ROX, Channels 1, 5, and 6 were empty. (b) Detection of HPV 18-ROX and HPV 31-FAM, Channel 2 empty. (c) Detection of HPV 33-FAM and HPV 45-ROX, Channels 4 and 8 empty.

The fluorescent reader used in this study has been developed further since the establishment of proof of principle on clinical specimens. Firstly, the fluorescent reader has been improved to detect multiple colours (Multiplex Detector Version 2). Detection of a second fluorescent colour doubles the number targets which can be identified on a single sample. Its performance was evaluated using the PreTect HPV-Proofer positive controls (Primer/Probe Mix: U1A-ROX, HPV16-FAM), (Primer/Probe Mix: HPV18-ROX, 31-FAM), (Primer/Probe Mix: HPV 33-FAM, 45-ROX), Figure 4. Acceptable sensitivity of the duplex detection was achieved and it is comparable with uniplex amplification. 

**Figure 5:**
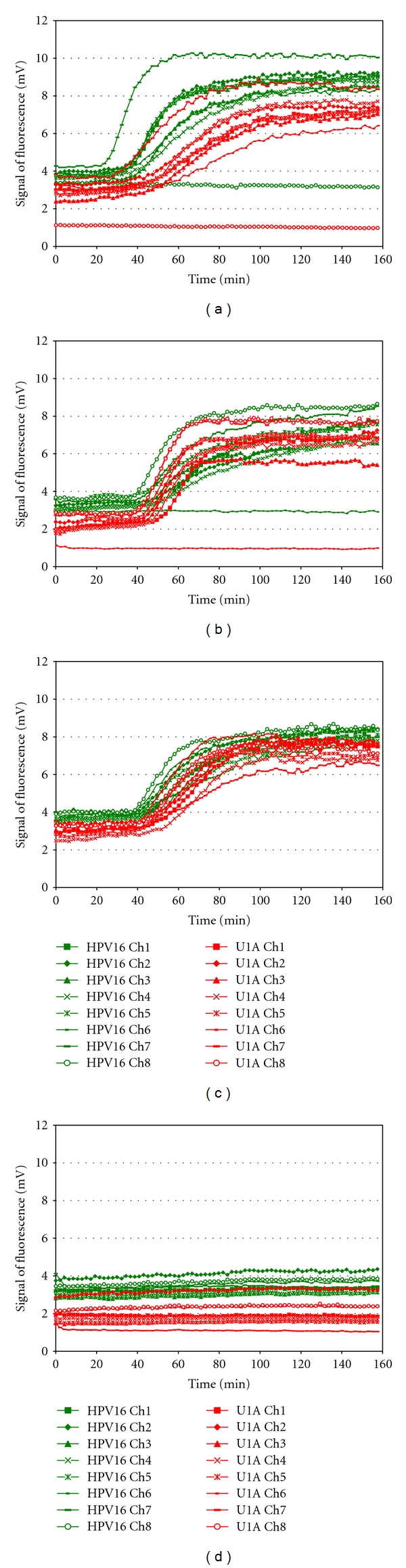
U1A and HPV16 amplification plots from NASBA chips containing freeze-dried reagents. Freeze-dried primers and probes (U1A/HPV16) were deposited in reaction chamber 1 (250 nL) and enzymes were deposited in reaction chamber 2 (125 nL). Incubation time in reaction chamber 1 was 5 minutes. The chips were stored at room temperature for 8 and 9 days. (a) Positive control U1A/HPV 16. Channel 8 was empty. (b) CaSki (1 : 10 dilution, equivalent to 370 cell/reaction chamber on chip). Channel 6 was empty. (c) HPV 16 positive clinical specimen (Pretoria). (d) No template control. Channel 7 was empty.

**Figure 6:**
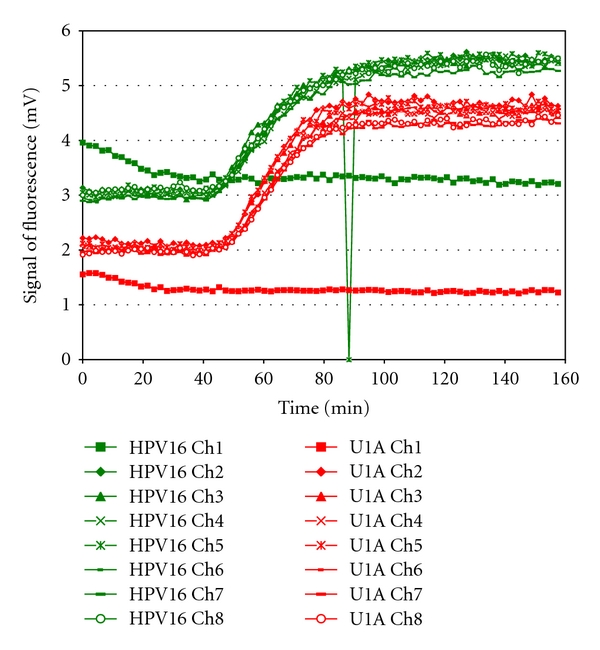
Amplification plot of HPV16 and U1A performed on the NASBA chip with freeze-dried enzymes in reaction chamber 2 stored at room temperature for 32 days. Channel 1 was empty. An instrumental error resulted in an outlier measurement for HPV16 in channel 5 after 88 minutes.

 In a second further development, some of the NASBA reagents were integrated into the NASBA chip. This was achieved by freeze drying of primer and probes and enzyme mix onto the chip as shortly described in materials and methods, a comprehensive description is published elsewhere [26]. For this experiment, we tested 4 chips including an HPV16/U1A positive control, CaSki cell mRNA, mRNA extracted from an HPV16 positive clinical specimen (from Pretoria Hospital) and a no template control (Figure 5). All channels which filled with reaction mix were positive for U1A and HPV16. The no template control was negative (Figure 5). Initial experiments have demonstrated that freeze dried enzymes can be stored on chip at room temperature with no loss of stability for at least 1 month (Figure 6).

## 4. Discussion 

This paper demonstrates the utility of a sample preparation microfluidic LOC device and associated instrument and a NASBA-based amplification chip with optical readers for sequential nucleic acid extraction and NASBA amplification of clinical material. The sample preparation device and a detailed description of the NASBA chip and reader has been described previously by the group [13, 24]. However, this paper describes further progress towards a “sample-in, answer-out” integrated microfluidic system tested on clinical samples. The proof of principle test identifies the challenges that must be overcome to achieve a fully integrated walk-away LOC system and demonstrates its potential utility with viscous and multicellular clinical specimens in a methanol-based cytology medium (PreservCyt). 

This development of a “sample-in, answer-out” chip achieves many of the goals of miniaturised LOC approaches and has the potential to offer a minimal handling, walk-away lab environment at an affordable price. In terms of the LOC environment, our approach to developing a “sample-in, answer-out” system has several potential commercial outcomes: including separate extraction and amplification on disposable chips, unified extraction and amplification, and an adaptable NASBA amplification platform for use in a range of disease analyses. 

The development of a fully functional integrated LOC microfluidic system that can perform cell lysis, nucleic acid extraction amplification, and detection with on-chip reagents is still a great challenge [2]; however, discrete examples of subfunctions of a complete system have been demonstrated in chip format over the last number of years and tested predominantly with cell line material, bacterial cultures, whole blood, or saliva specimens [27–30]. 

We have previously demonstrated successful amplification of the HPV targets of CaSki, HeLa, and MS751 HPV positive cell line mRNA prepared by the sample preparation platform to a detection limit of 5 cells, 5 cells and 50 cells, respectively, by PreTect HPV-Proofer [13]. In the present study, 20 clinical PreservCyt specimens were processed by the sample preparation platform and the performance of the combined sample preparation and NASBA amplification system was observed. The heterogeneity and varied cellular component of the test set of cervical PreservCyt specimens posed a serious challenge. To overcome this, an algorithm was developed in the LabView software to allow sequential loading of the cellular material to the cell capture filter membrane for cell lysis. For successful amplification of the nucleic acid extracted by the sample preparation device, dilution was necessary. The extraction chip produced nucleic acid of adequate quality for amplification in the NASBA reaction, with a detection rate of between 50–85%, depending on dilution of the extract from the LOC device. The requirement to dilute the eluate for successful NASBA following removal from the chip would indicate impurities in the nucleic acid preparation most likely related to incomplete evaporation of ethanol containing buffer from the SPE matrix and/or the retention of chaotropic salts from the lysis buffer on the membrane. Examples of this were samples 479 (HPV33+) and 522 (HPV16+) which achieved amplification in all channels at the 1 : 10 dilution but at the 1 : 5 gave 8 indeterminate results for HPV33 and 60% successful amplification for HPV16 at the 1 : 5 dilution, respectively. The requirement to dilute the nucleic acid was completely dependent on the quality of the individual sample extract, with one sample amplifying equally well at the 1 : 5 as at the 1 : 10 dilution (Sample 508: HPV33) and another sample giving consistently bad results at either dilution. Many studies including those describing LOC devices have highlighted the amplification problems associated with contaminating ethanol and salts [27, 28]. 

In this study, 16 NASBA chips were used to amplify nucleic acid from 6 specimens processed by the sample preparation platform which achieved positive amplification by PreTect HPV-Proofer and 9 NASBA chips were used in the optimisation of the freeze-dried reagents on control oligos. We have described previously the development of the NASBA platform [24] but have not demonstrated its capacity to amplify nucleic acids from either the sample preparation platform or clinical specimens until now. Overall, amplification efficiency for the NASBA chip was 69% (based on the number of positive reactions per channels filled on the chip), while the number of channels on the chips that filled correctly was 79.7% (premixed reagents). It is important to note that positive amplification of HPV mRNA was observed for each of the 6 clinical specimens tested. Amplification failure on chip, in channels that filled correctly, can be due to several reasons including: transfer of NASBA inhibitors from extraction chip elute and surface coating issues that result in a nonhydrophilic surface for NASBA reaction. 

Incomplete filling of amplification channels can result from failure of fluid progression through the microfluidic channel and clogging of the reaction channel at a number of critical stages in the chip assembly, including the bonding manufacturing process, bubble formation, the valve spotting procedure resulting in Teflon overflow, during the current manual sealing process or during the manual chip preparation process which involves application of a thin layer of grease to the chip to create an airtight seal between the chip and the instrument. The issue of Teflon overflow was a serious challenge and has since been eliminated by improving the spotting procedures, spotting accuracy, using more favourable valve geometry, and by optimizing concentrations of the hydrophobic valve solution [20]. It is not unlikely that the manual chip preparation procedures as well as the prototype nature of the instruments contribute to the observed malfunctions, and it should be possible to eliminate this by improving the chip fabrication and the fluidic interface between the instrument and the chip. It is important to note that the microfluidic yield of 79.7% (NASBA chips with premixed reaction mixture) and 83.3% (NASBA chips containing freeze dried reagents) is a highly encouraging result, especially taking into account the current manual procedures of lamination foil preparation, sealing and chip preparation prior to insertion into the instrument. This is the first study to comment on the performance of the microfluidic device that was developed therein on a number of clinical specimens. 

This paper presents predominantly a uniplex amplification detection approach. However, to demonstrate multiplexing feasibility, two spectrally resolvable dyes FAM and ROX have been successfully applied for duplex detection (Figures 4 and 5). Introduction of the duplex detection on-chip is an important improvement of the platform as it doubles the number of potential targets to be detected simultaneously on a single sample. The sensitivity of the duplex detection of different HPV types and testing amplification of freeze-dried reagents on-chip are acceptable and comparable with uniplex amplification. Testing for an increased number of targets on one and the same sample also reduces analysis costs. The use of specifically selected filters in the detection device allows separate detection spectra for the reporter molecules. The optical scanning device is spatially calibrated, allowing precise localisation of the detection chamber. Several detectors were originally evaluated and a detector based on best signal to noise characteristics and price was chosen. 

During the development of the NASBA LOC device, issues in relation to preloading of reagents (NASBA master mix, primers, probes, and enzyme) on chip became apparent. In order to achieve preloading of primers, probes, and enzyme a freeze-drying approach was adopted. This resulted in successful amplification of HPV16 and U1A in all channels which achieved sample filling (Figure 5). We are currently modifying the formulation of the NASBA master mix to achieve successful preloading of all reagents on chip. NASBA reagents such as DMSO and sorbitol not suitable for freeze drying will be stored on the sample preparation module as liquid (Figure 2(a)). Additionally, the next step involves preloading of all primers and probes mixes for the 5 HPV types and the U1A house keeping gene of the PreTect HPV-Proofer in the parallel reaction chambers on chip to achieve a complete “sample-in answer-out” point-of-care diagnostic platform for HPV. 

We have recently adapted the extraction and amplification chip for sequential integration. Test structures for chip to chip interface have been evaluated with promising results. The operating microfluidic principles of the two chips are different; in the sample preparation chip, the sample is pushed through the chip by pressure driven flow, while for the NASBA chip, capillary forces and pneumatic pressure are the actuation principles respectively. To ensure a leak-proof bond between the two chips, the connection point requires a gasket and air venting to remove the overpressure generated in the sample preparation chip before the eluate reaches the NASBA chip where capillary forces take over the actuation.

## 5. Conclusion 

The current paper describes a substantial advance on the state of the art for point-of-care HPV diagnostics technology. We presented a combined extraction and amplification in a microfluidic device consisting of extraction and NASBA chips. In addition, the adopted NASBA method offers the unique characteristics of isothermal amplification, greatly simplifying the thermocycling requirements for the system: effectively allowing a “blackbox” technology to be developed encompassing amplification and detection simultaneously in a real-time format. The amplification efficiency of the prototype NASBA platform has been compared to an industry gold standard for HPV detection with encouraging results. By this, we have demonstrated the subcomponents of a complete integrated in vitro diagnostic system: from clinical sample input to sample preparation to amplification to detection, thus advancing towards a “sample-in, answer-out” diagnostic platform. 

Finally, the technology platform is not limited to diagnostics of cervical precancer and cancer as studied in this work, but it has enormous potential in the monitoring and diagnosis of gene activity in areas such as infectious disease, oncology, immune response to allergens, immunotherapies, and chemotherapies.

## Figures and Tables

**Table 1:** NASBA chip results on clinical specimens. Six clinical specimens extracted on the sample preparation platform were tested on the NASBA chip platform for the dilutions 1 : 5 and 1 : 10. Positive amplification of target is denoted with “+”, while a negative result is denoted “−”. The table also shows the number of indeterminate reactions on chip, the number of channels filled and the overall amplification efficiency.

Sample			NASBA chip				
Clinical specimen ID	HPV type	Dilution of eluate	+	−	Indeterminate	No. channels filled	Overall [% positive] channels filled
478	HPV16	1 : 5	1	6	0	7	14
478	HPV16	1 : 10	0	7	0	7	0
479	HPV33	1 : 5	0	0	8	8	0
479	HPV33	1 : 10	8	0	0	8	100
508	HPV16	1 : 5	1	7	0	8	12.5
508	HPV16	1 : 10	0	0	0	0	0
508	HPV33	1 : 5	7	0	0	7	100
508	HPV33	1 : 10	7	0	0	7	100
511	HPV16	1 : 5	4	4	0	8	50
511	HPV16	1 : 10	6	2	0	8	75
520	HPV16	1 : 5	4	0	0	4	100
520	HPV16	1 : 10	5	0	0	5	100
522	HPV16	1 : 5	3	1	1	5	60
522	HPV16	1 : 10	8	0	0	8	100
522	HPV33	1 : 5	0	6	0	6	0
522	HPV33	1 : 10	0	6	0	6	0
